# Exercise program to reduce the risk of cognitive decline and physical frailty in older adults: study protocol for an open label double-arm clinical trial

**DOI:** 10.3389/fnagi.2023.1162765

**Published:** 2023-05-19

**Authors:** Moeko Noguchi-Shinohara, Kunihiko Yokoyama, Junji Komatsu, Kazumi Masuda, Mitsunobu Kouno, Mitsuhiro Yoshita, Kenjiro Ono

**Affiliations:** ^1^Department of Neurology, Kanazawa University Graduate School of Medical Sciences, Kanazawa, Japan; ^2^Department of Thyroidology, Public Central Hospital of Matto Ishikawa, Hakusan, Japan; ^3^Department of Preemptive Medicine for Dementia, Kanazawa University Graduate School of Medical Sciences, Kanazawa, Japan; ^4^Faculty of Human Sciences, Kanazawa University, Kanazawa, Japan; ^5^Faculty of Health Sciences, Kinjo University, Hakusan, Japan; ^6^Department of Neurology, National Hospital Organization, Hokuriku National Hospital, Nanto, Japan

**Keywords:** older adults, physical exercise, cognitive decline, physical frailty, mild cognitive impairment

## Abstract

**Objective:**

It is a big problem that many older adults are physically inactive. Well-known benefits of physical exercise include a decrease in the risk of cognitive decline and physical frailty. Therefore, this study aims to examine whether our proposed exercise program can prevent cognitive decline and improve physical function in the elderly.

**Methods:**

This study will include nondemented older adults (*n* = 160) without regular exercise habits. The trial will include a physical exercise training program (once a week) and nutritional lectures (once a month) over 5 months and follow-up for ≥1 year. The primary endpoint is the program’s efficacy in preventing cognitive decline, as assessed by changes in the memory performance index of the mild cognitive impairment (MCI) screen; the secondary endpoints are the incidence of MCI and dementia, physical testing, and frailty proportion. In the exploratory phase of the study, we will elucidate the underlying diseases causing MCI in community-dwelling older adults by neuroimaging.

**Discussion:**

This double-arm trial that aims to assess the impact of physical exercise on nondemented older adults’ cognitive and physical function. Furthermore, our newly developed exercise program will be easy for older adults to undertake.

**Clinical Trial Registration**: https://clinicaltrials.gov/, identifier [jRCT 1040220140].

## 1. Introduction

Alzheimer’s disease (AD) disease-modifying therapy can be useful, particularly during the mild cognitive impairment (MCI) and preclinical stages. Additionally, nonpharmacological approaches to MCI, such as lifestyle-based dementia prevention programs, including exercise and nutrition, can be efficacious for preventing or delaying the onset of dementia (Ngandu et al., 2015); to this end, early detection of MCI is essential. The prevalence of MCI among community-dwelling older adults has been reported at 17.0% (Ninomiya et al., 2020); however, the underlying diseases causing MCI are not sufficiently known because most patients with MCI do not go to the hospital or undergo either magnetic resonance imaging (MRI) or detailed cognitive tests.

Many older adults are physically inactive (Lee et al., 2012), although the health benefits of physical activity are well-known (World Health Organization, 2022). Physical exercise has been reported to reduce the risk of cognitive decline and physical frailty in older adults (Erickson et al., 2011; Cadore et al., 2014; Kulmala et al., 2014). In Japan, fractures and joint diseases, as well as dementia, are the two major diseases requiring nursing care for the elderly. If older adults would do a physical exercise routine, the number of older adults in long-term nursing care could decrease. However, in this country, only 44.4% of men and 37.1% of women ≥75 years old reported having a physical exercise routine (Cabinet Office, 2020). Therefore, an exercise program that would help older adults engage in a physical exercise routine is necessary.

Recently, we started a project for better diagnosing and preventing MCI among community-dwelling adults. A 10-min self-administered, electronically scored test called MCI screen (Cho et al., 2008; Shankle et al., 2009) is used to detect patients with MCI among nondemented elderly people. With respect to preventing dementia, we developed a physical exercise training program which is easy to start and continue for older adults, including multitasking, resistance, and aerobic exercises. We want to examine if the exercise program can reduce the risks of cognitive decline and physical frailty in this population.

The aims of this study are to examine whether the exercise program (i) can prevent cognitive decline and (ii) improve physical function and to elucidate the underlying diseases resulting in MCI among community-dwelling older adults.

## 2. Methods and analysis

### 2.1. Study design

**Figure 1:**
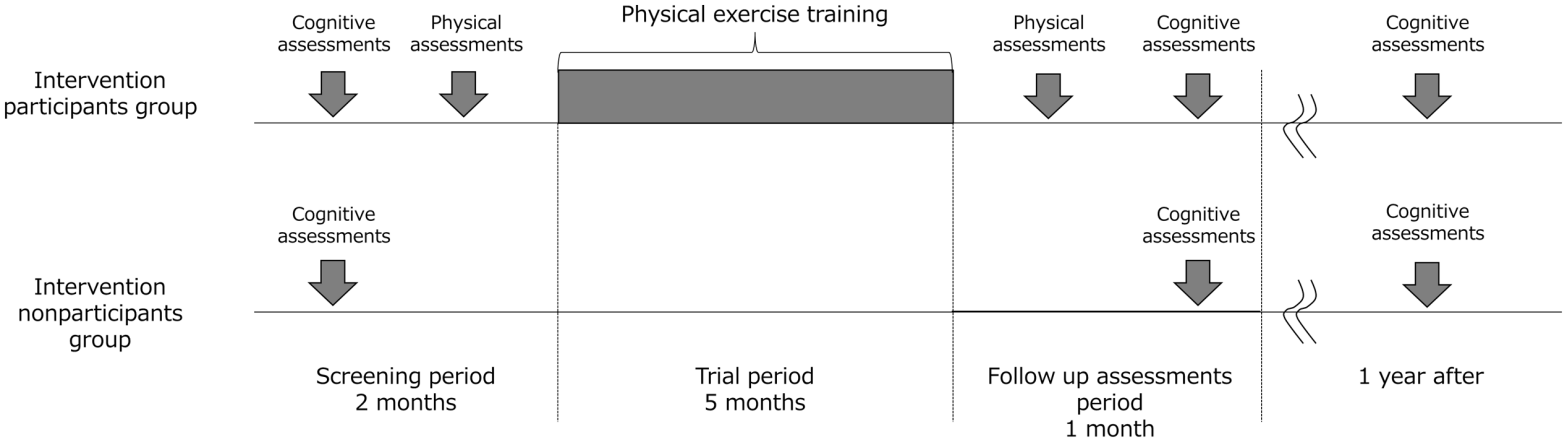
Study time course.

**Figure 2:**
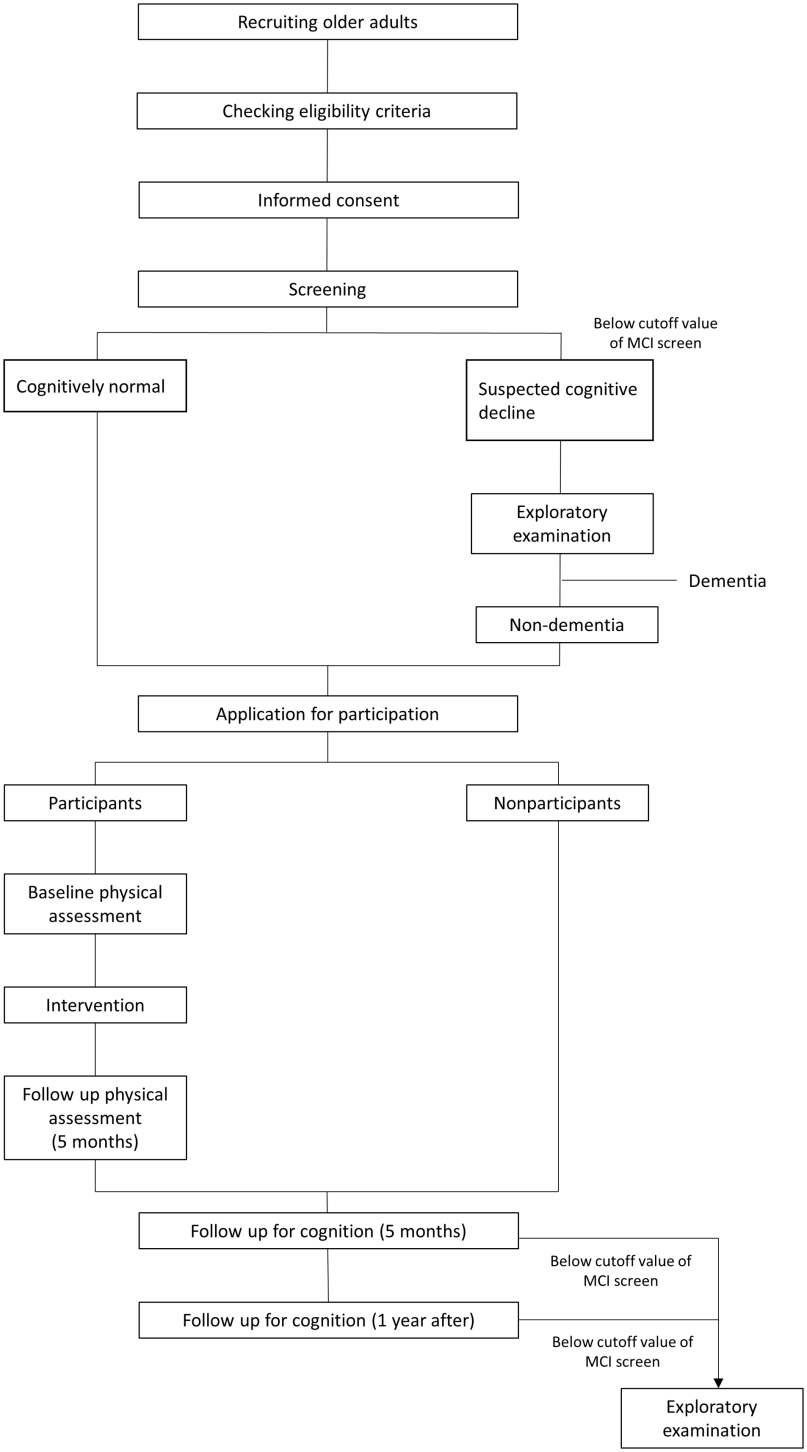
Flow chart of participants.

Nondemented older adults, including those with MCI (≥65 years) who do not have regular exercise habits will be recruited via local advertisements in Hakusan-city, Ishikawa Prefecture, Japan. This is an open label 1-year double-arm study that will be conducted in geriatric clinics and senior day care center in Japan. Figure 1 illustrates the study’s time course and Figure 2 shows flow chart of participants. Older adults will undergo the MCI screen which is used to detect patients with MCI, and all subjects with scores both above and below the cutoff of MCI screen except for subjects with dementia will be encouraged to participate in exercise classes.

### 2.2. Inclusion and exclusion criteria

The only inclusion criterium is age ≥ 65 years. The exclusion criteria include the following: (i) prohibition from exercising by a medical doctor, (ii) a diagnosis of dementia, and (iii) a nursing care level of the long-term care insurance (LTCI). In Japan, LTCI is a mandatory social insurance program that assists disabled older adults with daily living activities (Tamiya et al., 2011).

### 2.3. Intervention program

#### 2.3.1. Physical exercise training program

The intervention comprises individually tailored exercises, including multitasking, resistance, and aerobic exercises (Table 1). All training will be conducted at the gym once a week and guided by exercise therapists during the first 5 months. The resistance exercise program is standardized to include exercises for the six main muscle groups (quadriceps femoris muscle, hamstring muscle, latissimus dorsi and arm muscles, and abdominal and back muscles). The aerobic exercise program can be either cycling on an ergometer or treadmill running. The complete training program is supervised by professors of exercise physiology and exercise therapists.

**Table 1:** Physical exercise training program.

Multitask exercise		
	Exercise frequency per week	1
	Exercise duration (min)	15
Resistance exercise		
	Exercise frequency per week	1
	Exercise duration (min)	20
Aerobic exercise		
	Exercise frequency per week	1
	Exercise duration (min)	30

#### 2.3.2. Nutritional lectures

All participants will attend lectures on the principles of a healthy diet once a month during the first 5 months provided by the study nutritionist. The nutritionist will teach about a low-salt diet, diet to prevent diabetes and dyslipidemia, and protein intake to prevent frailty. Questionnaire on eating habit is obtained to assess the adherence to the proposal diet at baseline, the 5-month and 1-year follow-ups.

### 2.4. Screening, baseline, follow-up, and outcome measurements

Data will be obtained at four different times: at screening, at baseline, at the 5-month and 1-year follow-ups. The schedule for the variables to be measured is shown in Table 2. Cognitive test (MCI screen), physical test, body composition, questionnaire on nutrition/medical history/regular exercise will be performed at screening or at baseline and 5 months later. Additionally, the MCI screen, questionnaire on medical history/regular exercise will be obtained at 1-year visit.

**Table 2:** Schedule for the participants of the study.

	SC	Baseline	5-month	1-year
MCI screen	●		●	●
Physical test		●	●	
Body composition		●	●	
Nutrition questionnaire		●	●	
Questionnaire on medical history	●		●	●
Questionnaire on regular exercise		●	●	●

#### 2.4.1. Primary outcome measures

The primary outcome is the program’s efficacy in preventing cognitive decline, as assessed by changes in the memory performance index (MPI) scores of the MCI screen between the screening and 5-month and 1-year follow ups. We will compare between exercise participants and nonparticipants.

#### 2.4.2. Secondary outcome measures

Secondary outcomes include (i) the proportion of people who exercise regularly, (ii) the incidence of MCI and dementia at 1-year follow-up, (iii) changes in physical tests, (iv) body composition, and (v) the proportion of people who are frail and prefrail.

#### 2.4.3. Exploratory examination

For participants whose MPI is below the cutoff score at the screening or follow-up, a mini-mental state examination-Japanese (MMSE-J), brain MRI, ^123^I-iodoamphetamine (IMP)-cerebral blood flow single photon emission computed tomography (SPECT), and blood chemistry analysis [hemoglobinA1c, thyroid hormone, *Treponema pallidum* (TP) antigen STS test, TP antibody LA test, folic acid, vitamins B1 and B12] will be performed to elucidate the underlying disease of cognitive decline. Furthermore, subjects relevant to ≥1 of the following items are recommended for additional examinations: (i) low scores on the MMSE-J (<26 points) or zero points in the delayed recall sub-item, (ii) abnormality of disease-specific region on ^123^I-IMP-SPECT, such as the posterior cingulate, precuneus, and occipital lobe, (iii) hippocampal atrophy (>1.8 on the Voxel-based Specific Regional analysis system for Alzheimer’s Disease: VSRAD), (iv) severe leukoaraiosis. The additional examinations will include the Wechsler Memory Scale-Revised (WMS-R), ^11^C-Pittsburgh compound-B (PiB)-positoron emission tomography (PET), and ^18^F-fluorodeoxyglucose (FDG)-PET to diagnose the underlying diseases, including AD, vascular dementia (VaD), and Dementia with Lewy bodies (DLB).

We will investigate the relationships between program’s efficacy on cognitive function and amyloid pathology (positive or negative of PiB-PET) or arteriosclerotic factors (hypertension, diabetes mellitus, and dyslipidemia). We will also explore the association between the results of various examination such as brain MRI, ^123^I-IMP-SPECT, ^11^C-PiB, and ^18^F-FDG-PET and the progression of cognitive decline.

#### 2.4.4. MCI screen

The MCI screen requires 10 min; the MPI quantifies the pattern of recalled and nonrecalled words on a 0–100 scale, with lower scores indicating worsening cognition. It can distinguish healthy people from MCI with 96–97% accuracy (Shankle et al., 2009). We used a cutoff point of 50.2 to distinguish MCI.

#### 2.4.5. Diagnosis of MCI, dementia and its subtypes

MCI is defined by Petersen’s criteria (Petersen et al., 2001), and dementia is diagnosed according to the major neurocognitive disorder of Diagnostic and Statistical Manual of Mental Disorders, Fifth Edition (DSM-5) (American Psychiatric Association, 2013). In addition, to precisely diagnose MCI due to AD and AD dementia, we will use the National Institute on Aging and Alzheimer’s Association research framework (Jack et al., 2018). The diagnosis of other dementia subtypes will be made based on the National Institute of Neurological Disorders and Stroke-Association International pour la Recherche et I’Enseignement en Neurosciences criteria (Román et al., 1993) for VaD and the Fourth Consensus Report of the Dementia with Lewy Bodies Consortium (McKeith et al., 2017) for DLB. Clinical information, including neuroimaging findings, will be used for AD, VaD, and DLB diagnosis. Expert neurologists in the study team will adjudicate every case of dementia.

### 2.5. Assessment of physical frailty

Physical frailty and its phenotype will be assessed according to Fried criteria and the revised Japanese version of the Cardiovascular Health Study criteria (revised J-CHS criteria) (Fried et al., 2001; Satake and Arai, 2020), namely, weight loss, slowness, weakness, exhaustion, and low physical activity.

### 2.6. Sample size and statistical considerations

Assuming an alpha error of 5% (*α* = 5%) and a difference between intervention participants and nonparticipants groups exercise program values of *d* = 0.5, the required sample size for an 80% power is 64 participants per group. Considering a 20% expected loss to follow-up, the final sample size should be 160.

For comparisons between baseline and follow-up, repeated measures ANOVA will be used. The level of statistical significance will be 0.05. Data will be analyzed using the Statistical Package for the Social Sciences software (version 28; SPSS Inc., Chicago, IL, United States).

## 3. Discussion

Physical exercise is a promising intervention with strong potential to prevent or delay the onset of cognitive decline and physical frailty in older adults. Our study aims to assess the impact of a novel exercise program on older adults’ cognitive and physical function. We expect that older adults who complete the exercise program will show better cognitive and physical function than those who do participate in the exercise program. Moreover, we expect that our newly developed exercise program will be easy to start and continue for older adults. The one-year follow-up period may seem to short, however, it was reported that aerobic exercise training improved memory 6-months and 1-year after intervention (Erickson et al., 2011). Therefore, we believe we can achieve the primary outcome, which is the program’s efficacy on cognition, by comparing between exercise participants and nonparticipants in a1-year follow-up.

The multi-domain intervention studies have demonstrated mixed outcomes (Ngandu et al., 2015; van Charante et al., 2016; Andrieu et al., 2017). The large-scale open label, 6-year multidomain cardiovascular intervention trial with people aged 70–78 years old (*n* = 3,526) (van Charante et al., 2016), and the Multidomain Alzheimer Preventive Trial (MAPT) using polyunsaturated fatty acids, cognitive training, physical activity for 3 years with people aged 70 years old and over (*n* = 1,689) (Andrieu et al., 2017) both had no significant effects on cognitive decline; whereas the Finnish Geriatric Intervention Study to Prevent Cognitive Impairment and Disability (FINGER) trial demonstrated the effectiveness on delaying cognitive decline (Ngandu et al., 2015). This 2-year randomized controlled trial obtained 1,260 people with 60–77 years old. In our study, we will examine the effect of regular exercise on the prevention of cognitive decline.

The novelty of our study is to examine whether the exercise program is able to reduce the risk of cognitive decline in older adults with MCI due to AD according to biomarkers. We will investigate the association between AD biomarkers and progression of cognitive decline in this trial. High levels of cerebrospinal fluid (CSF) total tau levels and low levels of CSF amyloid-β_1–42_ were reported to be predictors of rapid cognitive decline in AD (Koch et al., 2013). In the Amyloid Tau Neurodegeneration (ATN) framework, amyloid positive in PiB-PET and low levels of CSF amyloid-β_1–42_ are amyloid markers, and AD findings of FDG-PET and hippocampal atrophy in MRI as well as high levels of CSF total tau are neurodegeneration markers. It was also reported the potential link between atherosclerosis and amyloid metabolism (Martorana et al., 2015). We will clarify the association between amyloid pathology, the presence of arteriosclerotic factors, and the exercise program’s efficacy in cognition.

A major limitation of this study is its nonrandomized control design. However, we hope to demonstrate that starting and continuing physical exercise can prevent cognitive decline and improve physical function in older adults. Additionally, we will elucidate the proportion of MCI due to AD with amyloid biomarker among community-dwelling older adults with MCI.

## 4. Dissemination

The results of our study will be presented in peer-reviewed journals. We have a plan to publish secondary analyses of our study and present at international conferences. We will also present the main results to our participants. For Original Research Articles, Clinical Trial Articles, and Technology Reports the Introduction should be succinct, with no subheadings. For Case Reports the Introduction should include symptoms at presentation, physical exams and lab results.

## Ethics statement

The research protocol was approved by the Ethics Committee of the Kanazawa University Medical Ethics Review Board (approval number 114156–1) and was conducted under the Declaration of Helsinki. All participants provided written informed consent for participation. The trial was registered, and a study protocol was uploaded to the Japan Registry of Clinical Trials with the identifier jRCT 1,040,220,140. The protocol employs relevant standard protocol items for clinical trials according to the CONSORT statement.

## Author contributions

MN-S, KY, KM, MK, and KO conceptualized the study and contributed to the study design. JK and MY collaborated to implement specific procedures. MN-S contributed to writing. KO was the principal investigator on the study, drafted the manuscript, and directed its implementation, including quality assurance and control. All authors read and approved the final manuscript.

## Funding

This work was partially supported by Grants-in-Aid for Scientific Research (Kakenhi) from the Japan Society for the Promotion of Science (JSPS) [grant number JP21K18425].

## Conflict of interest

The authors declare that the research was conducted in the absence of any commercial or financial relationships that could be construed as a potential conflict of interest.

## Publisher’s note

All claims expressed in this article are solely those of the authors and do not necessarily represent those of their affiliated organizations, or those of the publisher, the editors and the reviewers. Any product that may be evaluated in this article, or claim that may be made by its manufacturer, is not guaranteed or endorsed by the publisher.

## References

[ref1] American Psychiatric Association. (2013). Diagnostic and statistical manual of mental disorders, 5th ed. Washington, DC: American Psychiatric Association.

[ref2] Andrieu S. Guyonnet S. Coley N. Cantet C. Bonnefoy M. Bordes S. . (2017). Effect of long-term omega 3 polyunsaturated fatty acid supplementation with or without multidomain intervention on cognitive function in elderly adults with memory complaints (MAPT): a randomised, placebo-controlled trial. Lancet Neurol. 16, 377–389. doi: 10.1016/S1474-4422(17)30040-6 28359749

[ref3] Cabinet Office. (2020). Annual report on the ageing society.

[ref4] Cadore E. L. Moneo A. B. B. Mensat M. M. Muñoz A. R. Casas-Herrero A. Rodriguez-Mañas L. . (2014). Positive effects of resistance training in frail elderly patients with dementia after long-term physical restraint. Age (Dordr.) 36, 801–811. doi: 10.1007/s11357-013-9599-7 24243397 PMC4039260

[ref5] Cho A. Sugimura M. Nakano S. Yamada T. (2008). The Japanese MCI screen for early detection of Alzheimer’s disease and related disorders. Am. J. Alzheimers Dis. Other Dement. 23, 162–166. doi: 10.1177/1533317507312624, PMID: 18223126 PMC10846169

[ref6] Erickson K. I. Voss M. W. Prakash R. S. Basak C. Szabo A. Chaddock L. . (2011). Exercise training increases size of hippocampus and improves memory. Proc. Natl. Acad. Sci. U. S. A. 108, 3017–3022. doi: 10.1073/pnas.1015950108, PMID: 21282661 PMC3041121

[ref7] Fried L. P. Tangen C. M. Walston J. Newman A. B. Hirsch C. Gottdiener J. . (2001). Frailty in older adults: evidence for a phenotype. J. Gerontol. A Biol. Sci. Med. Sci. 56, M146–M157. doi: 10.1093/gerona/56.3.M146 11253156

[ref8] Jack C. R. Bennett D. A. Blennow K. Carrillo M. C. Dunn B. Haeberlein S. B. . (2018). NIA-AA research framework: toward a biological definition of Alzheimer’s disease. Alzheimers Dement. 14, 535–562. doi: 10.1016/j.jalz.2018.02.018, PMID: 29653606 PMC5958625

[ref9] Koch G. Belli L. Giudice T. Lorenzo F. D. Sancesario G. M. Sorge R. . (2013). Frailty among Alzheimer’s disease patients. CNS Neurol. Disord. Drug Targets 12, 507–511. doi: 10.2174/1871527311312040010 23574166

[ref10] Kulmala J. Solomon A. Kåreholt I. Ngandu T. Rantanen T. Laatikainen T. . (2014). Association between mid-to late life physical fitness and dementia: evidence from the CAIDE study. J. Intern. Med. 276, 296–307. doi: 10.1111/joim.12202, PMID: 24444031

[ref11] Lee I.-M. Shiroma E. J. Lobelo F. Puska P. Blair S. N. Katzmarzyk P. T. . (2012). Effect of physical inactivity on major non-communicable diseases worldwide: an analysis of burden of disease and life expectancy. Lancet 380, 219–229. doi: 10.1016/S0140-6736(12)61031-9 22818936 PMC3645500

[ref12] Martorana A. Di Lorenzo F. Belli L. Sacesario G. Toniolo S. Sallustio F. . (2015). Cerebrospinal fluid Aβ42 levels: when physiological become pathological state. CNS Neurosci. Ther. 21, 921–925. doi: 10.1111/cns.12476 26555572 PMC6493161

[ref13] McKeith I. G. Boeve B. F. Dickson D. W. Halliday G. Taylor J.-P. Weintraub D. . (2017). Diagnosis and management of dementia with Lewy bodies: fourth consensus report of the DLB consortium. Neurology 89, 88–100. doi: 10.1212/WNL.0000000000004058, PMID: 28592453 PMC5496518

[ref14] Ngandu T. Lehtisalo J. Solomon A. Levälahti E. Ahtiluoto S. Antikainen R. . (2015). A 2 year multidomain intervention of diet, exercise, cognitive training, and vascular risk monitoring versus control to prevent cognitive decline in at-risk elderly people (FINGER): a randomised controlled trial. Lancet 385, 2255–2263. doi: 10.1016/S0140-6736(15)60461-5, PMID: 25771249

[ref15] Ninomiya T. Nakaji S. Maeda T. Yamada M. Mimura M. Nakashima K. . (2020). Study design and baseline characteristics of a population-based prospective cohort study of dementia in Japan: the Japan prospective studies collaboration for aging and dementia (JPSC-AD). Environ. Health Prev. Med. 25:64. doi: 10.1186/s12199-020-00903-3, PMID: 33129280 PMC7603740

[ref16] Petersen R. C. Stevens J. C. Ganguli M. Tangalos E. G. Cummings J. L. DeKosky S. T. (2001). Practice parameter: early detection of dementia: mild cognitive impairment (an evidence-based review). Report of the quality standards Subcommittee of the American Academy of neurology. Neurology 56, 1133–1142. doi: 10.1212/WNL.56.9.1133 11342677

[ref17] Román G. C. Tatemichi T. K. Erkinjuntti T. Cummings J. L. Masdeu J. C. Garcia J. H. . (1993). Vascular dementia: diagnostic criteria for research studies. Report of the NINDS-AIREN international workshop. Neurology 43, 250–260. doi: 10.1212/WNL.43.2.250, PMID: 8094895

[ref18] Satake S. Arai H. (2020). The revised Japanese version of the cardiovascular health study criteria (revised J-CHS criteria). Geriatr Gerontol Int 20, 992–993. doi: 10.1111/ggi.14005, PMID: 33003255

[ref19] Shankle W. R. Mangrola T. Chan T. Hara J. (2009). Development and validation of the memory performance index: reducing measurement error in recall tests. Alzheimers Dement. 5, 295–306. doi: 10.1016/j.jalz.2008.11.001, PMID: 19560100

[ref20] Tamiya N. Noguchi H. Nishi A. Reich M. R. Ikegami N. Hashimoto H. . (2011). Population ageing and wellbeing: lessons from Japan’s long-term care insurance policy. Lancet 378, 1183–1192. doi: 10.1016/S0140-6736(11)61176-8, PMID: 21885099

[ref21] van Charante E. P. M. Richard E. van Dalen J. W. Ligthart S. A. van Bussel E. F. Hoevenaar M. P. . (2016). Effectiveness of a 6-year multidomain vascular care intervention to prevent dementia (preDIVA): a cluster-randomised controlled trial. Lancet 388, 797–805. doi: 10.1016/S0140-6736(16)30950-3 27474376

[ref22] World Health Organization. (2022). Global status report on physical activity. Available at: https://www.who.int/publications/i/item/9789240059153 (Accessed May 9, 2023).

