# Corrigendum: Exercise program to reduce the risk of cognitive decline and physical frailty in older adults: study protocol for an open label double-arm clinical trial

**DOI:** 10.3389/fnagi.2025.1573049

**Published:** 2025-03-10

**Authors:** Moeko Noguchi-Shinohara, Kunihiko Yokoyama, Junji Komatsu, Kazumi Masuda, Mitsunobu Kouno, Mitsuhiro Yoshita, Kenjiro Ono

**Affiliations:** ^1^Department of Neurology, Kanazawa University Graduate School of Medical Sciences, Kanazawa, Japan; ^2^Department of Thyroidology, Public Central Hospital of Matto Ishikawa, Hakusan, Japan; ^3^Department of Preemptive Medicine for Dementia, Kanazawa University Graduate School of Medical Sciences, Kanazawa, Japan; ^4^Faculty of Human Sciences, Kanazawa University, Kanazawa, Japan; ^5^Faculty of Health Sciences, Kinjo University, Hakusan, Japan; ^6^Department of Neurology, National Hospital Organization, Hokuriku National Hospital, Nanto, Japan

**Keywords:** older adults, physical exercise, cognitive decline, physical frailty, mild cognitive impairment

In the published article, there was an error in the section Methods and analysis, subsection *2.4.4. MCI screen*, paragraph 1, in which the reference “Shankle, W. R., Mangrola, T., Chan, T., and Hara, J. (2009). Development and validation of the memory performance index: reducing measurement error in recall tests. *Alzheimers Dement*. 5, 295–306. doi: 10.1016/j.jalz.2008.11.001” was erroneously replaced with the reference “Cadore, E. L., Moneo, A. B. B., Mensat, M. M., Muñoz, A. R., Casas-Herrero, A., Rodriguez-Mañas, L., et al. (2014). Positive effects of resistance training in frail elderly patients with dementia after long-term physical restraint. *Age (Dordr.)*. 36, 801–811. doi: 10.1007/s11357-013-9599-7”.

The corrected sentence appears below:

“It can distinguish healthy people from MCI with 96–97% accuracy (Shankle et al., 2009).”

In the published article, there was an error in the Abstract in the description of sample size number. This sentence previously stated:

“This study will include nondemented older adults (n = 103) without regular exercise habits.”

The corrected sentence appears below:

“This study will include nondemented older adults (*n* = 160) without regular exercise habits.”

The authors apologize for these errors and state that they do not change the scientific conclusions of the article in any way. The original article has been updated.

